# In Situ Raman Spectroscopy for Early Corrosion Detection in Coated AA2024-T3

**DOI:** 10.3390/s25010179

**Published:** 2024-12-31

**Authors:** Adrienne K. Delluva, Ronald L. Cook, Matt Peppel, Sami Diaz, Rhia M. Martin, Vinh T. Nguyen, Jeannine E. Elliott, Joshua R. Biller

**Affiliations:** 1 TDA Research Inc., Golden, CO 80403, USA; adelluva@tda.com (A.K.D.);; 2 Molecular Design Innovations, LLC, Golden, CO 80403, USA; 3 Medtronic Inc., Louisville, CO 80027, USA

**Keywords:** aluminum, metal coatings, EIS, Raman spectroscopy, acid corrosion, pitting corrosion

## Abstract

Here we describe the synthesis and evaluation of a molecular corrosion sensor that can be applied in situ in aerospace coatings, then used to detect corrosion after the coating has been applied. A pH-sensitive molecule, 4-mercaptopyridin (4-MP), is attached to a gold nanoparticle to allow surface-enhanced Raman-scattering (SERS) for signal amplification. These SERS nanoparticles, when combined with an appropriate micron-sized carrier system, are incorporated directly into an MIL-SPEC coating and used to monitor the process onset and progression of corrosion using pH changes occurring at the metal–coating interface. The sensor can track corrosion spatially as it proceeds underneath the coating, due to the mobility of the proton front generated during corrosion and the homogeneous distribution of the sensor in the coating layer. To our knowledge, this report is the first time a 4-MP functionalized gold nanoparticle has been used, along with SERS spectroscopy, to monitor corrosion in an applied commercial coating in a fast, non-contact way.

## 1. Introduction

Corrosion is a costly problem that impacts the safety and sustainment of metal components and structures. Specifically in the aerospace industry, civilian and military aircraft are at risk of catastrophic failures when corrosion-causing conditions (moisture, salt, and heat) degrade critical parts [1,2]. Coatings are applied to protect metal surfaces and prolong service lifetimes; however, these coatings are opaque and block visible inspection of early-stage corrosion on metal surfaces which could be used to direct corrosion mitigation. Utilizing a “smart” coating that can self-report corrosion occurring on the metal surface underneath is a promising strategy to accomplish this [3]. Smart coatings could allow for the collection of performance data on aerospace alloys under real-world corrosion conditions to guide future design. One of the key advantages to using a coating-based solution, as opposed to macroscale sensors like optical fiber sensors, is that corrosion can be detected anywhere that the coating is, instead of only in the location where the macroscale sensor was installed [4,5].

**Figure 1:**
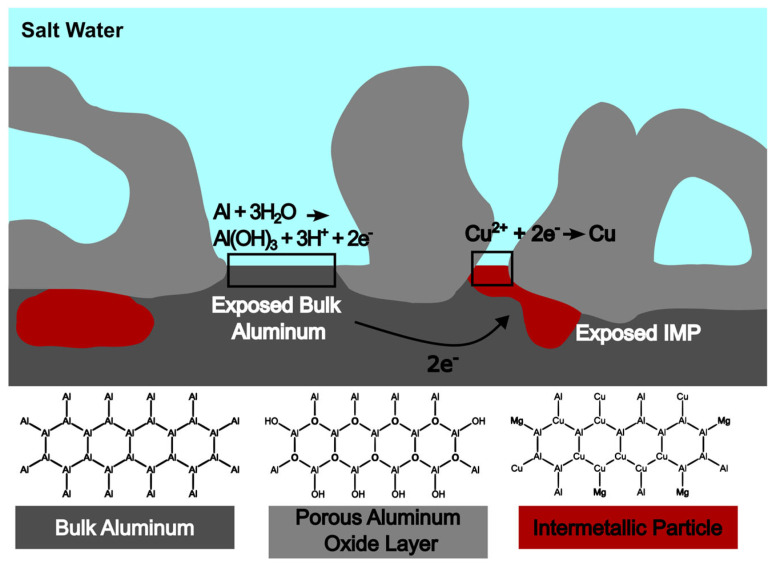
Illustration of corrosion occurring near the metal surface on AA-2024. An acidic environment is a hallmark of severe corrosion.

Corrosion is an electrochemical reaction involving the transfer of electrons between a metal surface and electroactive compounds in solution (Figure 1). During corrosion in aerospace grade aluminum alloys like AA2024, the oxidation of the aluminum (anode) produces protons and the reduction of oxygen (in the presence of water) forms hydroxide (cathode). The corrosion of metals is electrochemically driven by the two half-cells:

Cathodic: ½ O_2_ + H_2_O + 2e^−^ → 2OH^−^ (basic)

Anodic: Al → Al^3+^ + 3e^−^ + 3H^+^ (acidic)

At the anodic half-cell, metals lose electrons and typically form soluble metal complexes with water (or chloride anions if there is salt around) and, in addition, make the water around the corrosion site more acidic (more H+). The pH value, especially the acidic pH values, have been well correlated with different corrosion rates in the literatures [6,7]. Corrosion half-cells are coupled to each other and the electrons from the anodic half-cell are transported through the metal to the cathodic half-cell. The electrons supplied to the cathodic half-cell are used in cathodic reduction reactions. Typically, the cathodic reaction is the reduction of oxygen to hydroxide which makes the water surrounding the cathodic half-cell more basic [8,9].

There is a brief initial period of corrosion in AA2024 where the intermetallic phase is anodic with respect to the bulk aluminum. This causes the intermetallic phase to oxidize which releases copper that quickly redeposits on the aluminum to form copper islands with high surface areas. It is the oxygen reduction at these copper islands that forms the new cathodic half-cells and drives the pitting (anodic corrosion) of the bulk aluminum [10]. Thus, after the initial corrosion phase, the main ongoing corrosion products are aluminum ions, protons, and hydroxide. The corrosion products of protons and hydroxide ions are not exclusive to corroding aerospace aluminum. The electrochemical reactions for corrosion in steel are similar, where the anodic metal dissolution is from solid iron to Fe^2+^. The production of H+ and OH- is fundamental to corrosion in both aerospace-grade aluminum and steel alloys, which are more routinely deployed in ground-based vehicles.

### 1.1. Raman Spectroscopy and Surface-Enhanced Raman Scattering (SERS)

When incident light interacts with matter (i.e., molecules), the light is scattered either elastically (Rayleigh scattering) or inelastically (Raman scattering). Raman scattering can occur with much a lower amplitude than other optical phenomena like fluorescence, depending on the material being analyzed. Nonetheless, since the scattering of the incident light is directly tied to the chemical bonds in the molecule being measured, Raman spectroscopy can yield highly specific information about a specific chemical environment. Raman spectroscopy has been used in the field of corrosion before, primarily to identify and characterize corrosion products and inhibitors in a laboratory setting [11,12].

Raman spectroscopy is aided routinely aided by a signal amplification scheme called surface-enhanced Raman scattering (SERS). SERS was first observed in 1973 on the surface of a silver electrode [13], and is now commonplace as a method of amplifying signals in Raman spectroscopy. SERS uses a rough metal (gold, silver, or copper) surface to amplify the signal of a Raman-active species absorbed onto it, and has been shown to enhance the signal of a molecule by over 1,000,000 times [14]. The physics underpinning the enhancement of SERS have been described in extensive detail [15,16], pertaining to the enhancement of the incident light at the metal surface through the creation of surface plasmons. These surface plasmons induce a stronger spatial polarization of the incident light than would have otherwise been experienced by the molecule in the absence of the metal surface, which results in a much stronger amplitude signal.

The SERS spectroscopy technique has been used by many researchers to measure pH in biological and wearable sensor applications [17,18]. Though first developed on flat metal surfaces, SERS has been increasingly used with metal nanoparticles which are functionalized with one or more probe molecules. The SERS effect can vary extensively depending on the size of the metal nanoparticles used [19,20], as well as on the identity of the nanoparticle (usually silver or gold [21]). Metal nanoparticles have given way to nanostructures with vertices and gaps, which further enhance the SERS response [22]. There has been a wide effort to develop and characterize nanoparticle-based SERS in a solution, but to our knowledge, our work is the first to report nanoparticle-based SERS being deployed in a cured epoxy primer on a metal surface.

### 1.2. 4-Mercaptopyridine as a Window into pH of the Local Environment

The molecule attached to the gold nanoparticle, 4-mercaptopyridine (4-MP), is well established for sensing pH changes in a solution as part of a SERS approach [23]. Recently, SERS-amplified 4-MP has been used to monitor the pH microenvironment related to cancer state [24] and attached to functionalized gold nanostars to monitor pH in relation to food freshness [25]. In one report, bulk pH measurements of semi-solid preparations were taken by functionalizing immobilized gold nanoparticles with 4-MP. In this configuration, and in the semi-solid mixtures tested, 4-MP was reported to have the clearest change in Raman peak shifts in the range of pH 3–10 [26]. Within the past year, 4-MP attached to gold nanoparticle functionalized electrodes was used to reveal the electrolyte pH values in the protonation/deprotonation process of 4-MP, which was correlated to the adsorption mode (inclined or vertically) of the molecule to the gold surface [27].

We chose to specifically track the ratio of changes in the peaks at 1580 cm^−1^ and 1610 cm^−1^ to gauge the pH response of the molecule, over other peaks such as the one at 1000 cm^−1^. As described in Wattanavichean et al. [28], the molecular motion assigned to 1000 cm^−1^ is a ring breathing mode, while the Raman peaks at 1580 cm^−1^ and 1610 cm^−1^ are directly related to the unprotonated or protonated state of the pyridine ring, respectively. For this reason, we selected the 1580 cm^−1^/1610 cm^−1^ pair. We also note that once the 4-MP functionalized nanoparticles were attached to the carrier and dispersed in the epoxy coating, the 1580 cm^−1^/1610 cm^−1^ responded to pH changes in a much more repeatable fashion compared to the peak at 1000 cm^−1^.

The use of these two peaks in aqueous solutions has been well characterized in the literature, and this particular region changes in response to pH at multiple excitation wavelengths from 514.5 nm–1064 nm [29]. The work we report on here is the first to demonstrate that the same peaks can be monitored for pH in a coating system. When deprotonated (high pH), the 1580 cm^−1^ peak is larger, and when protonated (low pH), the 1610 cm^−1^ peak is larger. Thus, pH can be tracked by using the ratio between the intensities of these two peaks. We call this the “peak Raman ratio”, or PRR, throughout this paper, defining it as $PRR=\frac{I_{1580}}{I_{1610}}$, making larger PRR values correspond to higher pH values and smaller values correspond to lower pH values. Throughout the paper, data will be presented in terms of the % change from the original PRR measurement of a sample.

### 1.3. Existing State of the Art in Smart Coatings for Corrosion Monitoring

Characterization of these molecular sensing additives in a real coating system is not common. Often, additives and pigments are included in aerospace coatings that conflict with the optical responses of the molecular sensors, where the sensor might work perfectly in a clear epoxy but cannot be seen in an opaque primer. Coatings utilizing molecular-scale sensors often depend on a change in the optical response of a probe molecule when one or more products of the family of corrosion chemical reactions are present. An early example of this approach is a smart coating with pH-triggered release microcapsules used to visually indicate early-stage corrosion developed by NASA’s Corrosion Technology Lab [30]. Molecular scale sensors can also be based on color change in the visible spectrum [31,32] and also through pH (or metal ion) activation of fluorescent sensors [33,34]. In another report, pH-sensitive fluorescent probes incorporated into silica nanorods were designed to detect the cathodic (OH-producing) half of the corrosion reaction [35].

Raman spectroscopy related to corrosion research has previously centered on studying the binding activity of corrosion inhibitors. A summary of several of these types of studies is provided in a review by Frank et al. [36]. Michailidou et al. detailed the use of Raman spectroscopy to determine the composition of the protective layer formed on AA2024-T3 and AA2198-T8 under acidic conditions and in the presence of lithium carbonate [37]. This study, while interesting, was performed with only an electrolyte solution above the panel and did not deal with the complexity of SERS spectroscopy in a commercial epoxy primer system. An overview of all corrosion monitoring techniques ca. 2023 is given by Rajendran [38]. Monitoring pH is an established way of tracking corrosion progress, but to date all such measurements have required physical electrical connections into the sample being tested.

The SERS/4-MP combination has been applied widely across many different applications, and tracking pH as an indicator of corrosion by electrically connecting to the sample is commonplace. However, this report is the first, to our knowledge, to describe 4-MP functionalized gold nanoparticles that report on pH/corrosion in situ in a non-contact way via Raman spectroscopy to read out changes from a commercial epoxy coating in contact with corroding aluminum underneath.

## 2. Materials and Methods

### 2.1. Corrosion Sensor Synthesis

A full description of the sensor synthesis is given in [39]. A brief description is given here. All reagents were purchased form MilliporeSigma (St. Louis, MO, USA) and used as received. The first step was to synthesize the gold nanoparticles following the procedure in Xu et al. [40]. The 4-MP was added in as a 5 mM aqueous solution to the gold nanoparticle colloid. This was then allowed to stir for 5 min. An alumoxane carrier particle was necessary to disperse the 4-MP functionalized nanoparticle into the primer. The carrier particle was synthesized as described in [39] and mixed together with the 4-MP functionalized gold nanoparticle solution. The gold colloid solution is initially purple, but the solution loses this color and begins to clear as the colloid is mixed with the carrier. Conversely, the carrier powder is white at the start but turns purple as the gold colloid attaches to the surface of the carrier particles. Afterwards, the powder was centrifuged and dried and could then be incorporated into the primer with simple mixing.

### 2.2. Particle Characterization

Nanoparticle colloids were characterized prior to adding the 4-MP and carrier particles in order to assess the AuNP size. A Nanotract single channel DLS instrument was used and all particle statistics were calculated using the intensity data. Scanning electron microscopy (SEM) was used to image the sensor powder once fully synthesized. A Hitachi TM4000PlusII tabletop scanning electron microscope (Hitachi High Tech Group, USA) was used in backscatter mode at a 15 kV operating voltage. This machine was also equipped with EDS and was operated at 20 kV for EDS analysis. The samples were coated in a 3.5 nm platinum coating prior to imaging to enhance their conductivity.

### 2.3. Test Panel Preparation

All experiments were performed using the aluminum alloy AA2024 with T3 temper, unless otherwise specified. Metal sheets were cut to any of the following specifications, depending on the test: 3″ × 6″ × 1/32″, 12″ × 12″ × 1/32″, 3″ × 3″ × 1/32″, or 1″ × 4″ × 1/8″. If using an alodine pretreatment, panels were sent out to Automatic Anodizing (Chicago, IL, USA) where the alodine 1200 chemical conversion coating was applied according to the manufacturer’s specifications. Test panels were coated in Deft^®^ MIL-DTL-53030 water reducible epoxy primer (PPG, obtained from SkyGeek (LaGrangeville, NY, USA). Mixing and formulation followed the ratios on the specification sheet. The sensor was added in at varying weight percentages to the “part B” coating component (the one without added solids), then mixed thoroughly in a speed mixer (FlackTek, Landrum, SC, USA) with a mixing media for 2 min prior to combination with the remaining coating parts. Induction times were observed in accordance with the coating specification sheet. Admixed resin was sprayed onto the AA pieces using a high-volume low-pressure spray gun operated in the pressure range specified by the coating specification sheet. The panels were scrubbed with a 3M abrasive pad and solvent washed immediately prior to spraying out to remove any surface oxidation and promote adhesion. If included in the test panel design, panels were scribed using a Dremel tool or bolted with cadmium-plated screws. All coatings were sprayed out to the MIL-SPEC thicknesses, which were confirmed using a DeFelsko thickness gauge. Panels were allowed to cure in ambient conditions for 72 h before any testing.

### 2.4. Raman Spectroscopy

The coated panels were tested using a TSI Chemlogix portable EZ Raman NP spectrometer (Shoreview, MN, USA, 785 nm laser wavelength) with a standoff distance of 7 mm. This distance was consistently achieved using a rubber bumper. Measurements were taken using a laser power of 100 mW/cm^2^ and measurement times between 5 and 30 s, depending on the sample. At least 3 measurements were taken of each sample in different locations, then averaged together. The autobaseline feature within the instrument was used for background corrections.

### 2.5. pH Measurements

Acetic acid or 20% sodium hydroxide solutions were diluted with DI water to achieve specified pH values in the range of 1–12. The pH values were measured using an Oakton pH probe, then remeasured before every experiment. Approximately 0.2 wt.% of the sensor was dispersed into a pH solution for the solution calibrations. For the panel pH calibration, approximately 2–3 mL of pH solution was pipetted onto a coated panel (0.5 wt.% sensor) surface, lying flat. Once the solution had been absorbed into the panel coating, Raman measurements were taken on the spots where the solution had been deposited. No significant signal difference was seen between the slightly damp panel and the fully dry panel.

### 2.6. Electrical Impedance Spectroscopy (EIS)

The 3″ × 3″ panels coated in primer were clamped underneath 0.5% NaCl solution to drive accelerated corrosion. At each measurement time interval, the panel was removed from the 0.5 M NaCl solution and EIS was performed using a saturated KCl electrolyte solution. After EIS was complete, the panels were clamped back beneath the 0.5% NaCl until the next test point. EIS data were collected from 5000 Hz to 0.025 Hz. EIS data were fitted to a simple model with inputs comprising the resistance of the solution (R_soln_), the coating porosity (R_po_), a double-layer capacitance (C_dl_), coating capacitance (C_coat_), and the charge transfer resistance of the corroding system (R_CT_). The fitting of the data was performed using Gamry’s Echem Analyst software (version 6.33.4707) with a simplex optimization method.

### 2.7. Salt Fog Testing

Two Q-lab (Westlake, OH, USA) Q-FOG corrosion testing chambers were used for accelerated environmental corrosion exposure. Model SSP was used to run ASTM B117, which is a constant salt fog (5% NaCl solution) kept at 35 °C. Model CCT was used to run the GMW14872 24 h cycle, which includes the following steps: (1) 8 h at 25 °C, 45% relative humidity, and intermittent salt spray; (2) 8 h at 49 °C and 100% relative humidity, (3) 8 h at 60 °C and ≤30% relative humidity. All test panels were placed in the chamber at a 20-degree angle to allow for saltwater runoff.

### 2.8. Coating Removal

Chemical paint strippers were used to remove the coatings after corrosion testing without disturbing any of the corrosion damage on the underlying panel surface. PPG PR-3500 was used, purchased from SkyGeek. Full panels were left in a bath of the stripper for the length of time required to remove the coating (anywhere from 30 min to 24 h, depending on the coating stack up and the degree of corrosion exposure). After the stripper bath, the panels were removed and scraped off using a plastic scraper, then rinsed with water and dried. Only panels with the same coating and sensor type were left in the stripper bath together.

### 2.9. Visualization

A Keyence red laser profilometer was used to observe the panel surface condition, with images taken of any corrosion damage. Images were taken both optically and with the red laser (2.5–50X) to characterize damage features such as pitting and salt deposits within the scribe. The dimensions of such features were measured. The profilometer was also used at 10–100X magnification to image the sensor powder particles and assess their morphology and average size.

### 2.10. Physical Property Testing

Standard testing was performed to assess the physical properties of the primer coatings after the coatings had cured on the test panels. Primer-only, non-pretreated test panels were used. Specific tests, which included coating adhesion, color, solvent resistance, flexibility, and hardness, were taken directly from MIL-DTL-53030D, closely following the specifications outlined in the document, and from the referenced ASTM standard test method. All tests compared the performance between samples with and without the sensor, from the same batch of primer. A summary of the referenced methods is shown in Table 1. Results of physical property testing are shown in (Supplementary Table S1).

## 3. Results

### 3.1. Characterization of Sensing Particles

The full corrosion sensor synthesis began with creation of the gold nanoparticles required for signal enhancement through SERS. Dynamic light scattering (DLS) was used to characterize the size of gold nanoparticles. The mean diameter of the area distribution (MA) was measured to be 99.8 nm ± 2.6 nm with a PDI of 0.108 ± 0.030 (Supplementary Figure S1). Once the gold-nanoparticles were synthesized, they were combined with 4-MP. The 4-MP functionalized gold nanoparticles were then loaded into the alumoxane carrier particles, which are on the scale of 5 µm. We refer to the fully constructed sensor as the “powder sensor”, as it functions as any other drop-in inorganic additive used in coating systems.

**Figure 2:**
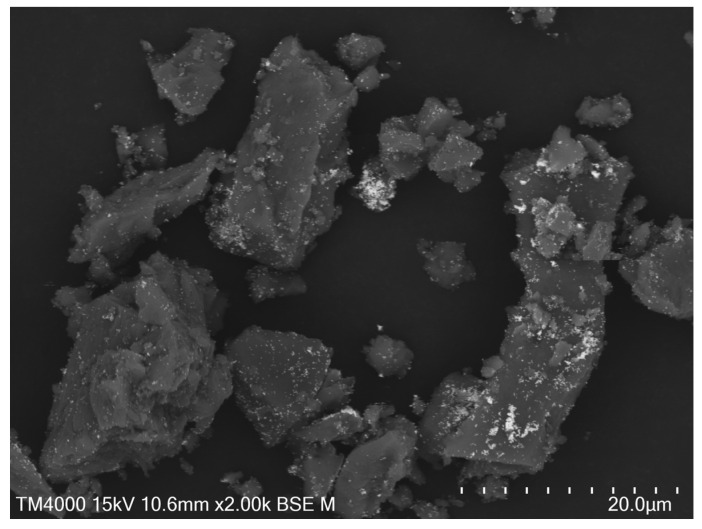
SEM of sensor powder in backscattering mode: bright spots are gold.

Scanning electron microscopy (SEM) of the powder sensor, once fully synthesized, was undertaken to assess the overall morphology (Figure 2). The scans were taken in BSE mode to enhance the contrast between the gold nanoparticles and the ceramic carrier particles. EDS was used to confirm that the bright spots were gold (Supplementary Figure S2). A wide variety of carrier particle sizes could be seen, ranging from 5 to 20 μm. The particle morphology is highly rough and angular, likely a result of the grinding procedure and the brittle nature of the material. Bright, gold spots can be seen covering the carrier particles, confirming gold nanoparticle adsorption onto the carrier. The distribution is not perfectly even, as some spots where the gold nanoparticles formed large clusters can be seen.

**Figure 3:**
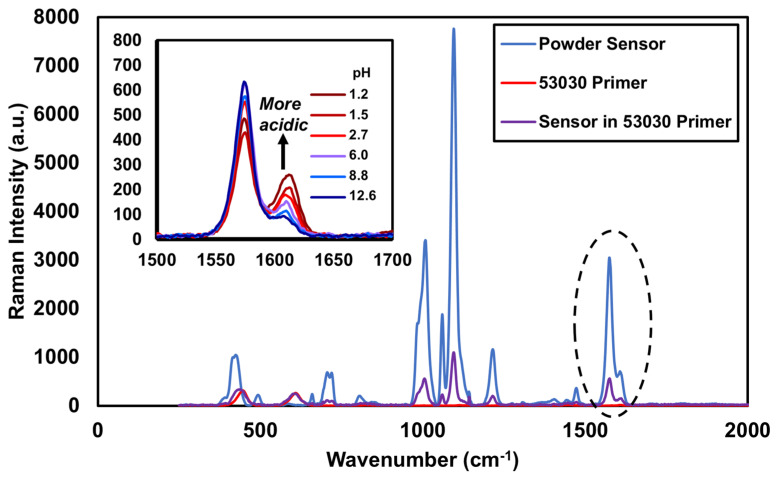
Raman spectrum of powder corrosion sensor loaded into a primer at 150 ppm, compared to the Raman spectra of the primer alone and the powder sensor alone. (Inset) The Raman spectra of the primary peaks of interest for 4-MP as a function of changing pH, from 1.2 to 12.6.

The presence of 4-MP on the gold nanoparticle surfaces was confirmed through Raman spectroscopy of the powder sensor (Figure 3). The signal was very high as a result of the SERS effect, indicating successful adsorption of the 4-MP onto the gold nanoparticle substrate. We were not able to quantify the signal enhancement, as without gold nanoparticles present to facilitate SERS, no Raman signal can be seen from the 4-MP. We note that based on the SEM, the density of gold nanoparticles is quite low. This indicates that the bulk of the SERS enhancement is coming from the surface structure of individual nanoparticles, rather than plasmon resonance “hotspots” created in between the gold nanoparticles. This suggests that the signal enhancement achievable with this approach could be orders of magnitude higher if the density of gold nanoparticles on the carrier can be increased.

The powder sensor was then added into a primer coating and the Raman spectrum was compared to that of the pure sensor, as well as the neat primer (Figure 3). Overall, the spectrum of the sensor in the primer looks like the addition of the primer spectrum and the powder spectrum. All primary peaks of the 4-MP molecule are prominent once incorporated into the primer. The signal intensity is lower, however, due to the low percentage of the sensor loaded into the primer (150 ppm). The primary Raman peaks in the primer spectrum are well separated and come from the TiO_2_ at 445 and 610 cm^−1^ and the 4-MP.

### 3.2. Validating Sensor pH Response

**Figure 4:**
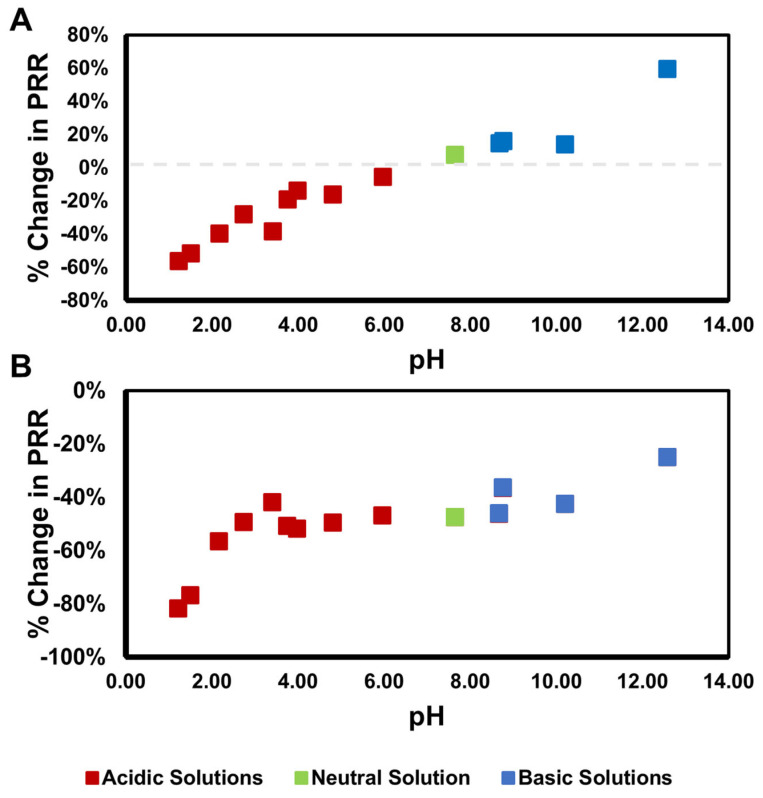
Percentage change in PRR for (**A**) the sensor dispersed in aqueous solution as a function of pH and (**B**) loaded into the primer coating, with pH solution applied to the coatings. Both graphs share the same legend.

Because the corrosion sensing system presented here is a pH sensor, the first test was performed with solution-based measurements to confirm the capabilities of the sensor. The sensor powder was dispersed into aqueous solutions with pHs varying from 1 to 12. Following that, the pH-sensing capabilities were tested with the sensor dispersed into a primer coating, with the same pH solutions applied atop. The PRR was recorded for each sample and is presented as a percentage decrease from the “standard” sample—the powder sensor was used for the solution measurements and a coated panel without any pH solution was used for the panel measurements. The PRR change is plotted in Figure 4 for both sets of experiments as a function of pH.

The neutral pH solution corresponds with that of the as-made powder, with a relative change of approximately 0%. When the pH decreases, the 1608 cm^−1^ peak increases, which in turn decreases the PRR, following a linear trend. When the pH increases, the opposite occurs and the PRR increases (Figure 3). When looking at the panel data, the behavior changes. The environment surrounding the dispersed sensor in the epoxy primer is much more complex than water. As such, the “baseline” spectra are different—that is, the sensor is experiencing a more acidic environment when distributed in the primer than when analyzed in the powder form. When the pH solutions are added, the PRR decreases at very low pH values (pH 1–3) and is relatively constant between pHs 4 and 9, then increases from pHs 9–13.

### 3.3. Measuring Coating Physical Properties with Corrosion Sensor Mixed in

The corrosion-sensing additive was designed to be incorporated into existing military-qualified coating systems. Physical property testing of the coating was performed, with and without the sensor, to ensure that the sensor does not degrade any of the specifically engineered physical properties of the coating. A representative set of tests (Table 1) were chosen from the list of tests outlined in the MIL-DTL-53030 specification document. The primer performed the same in both conditions, unmodified or with the sensor, and the results were within spec. The sensor did not degrade the performance of the primer, nor did it interfere with the curing of the epoxy primer.

### 3.4. Detection of Corrosion on AA2024 Flat Panel with Corrosion “Smart” Paint

**Figure 5:**
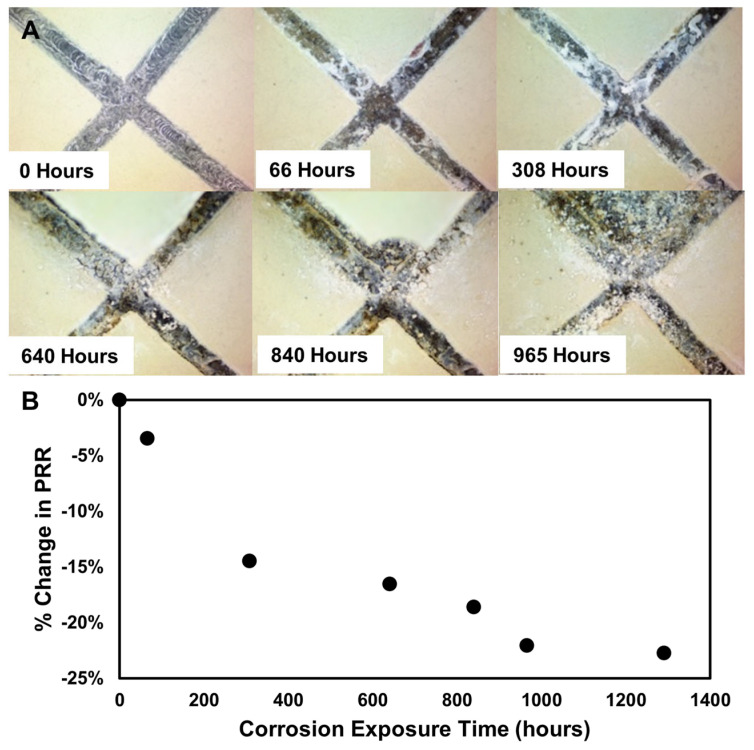
Corrosion exposure results for a scribed AA-2024 panel coated in MIL-DTL-53030 primer, loaded with 150 ppm of corrosion sensor. (**A**) Photos of the scribe center as a function of time. (**B**) Percentage change in the PRR as a function of time.

The corrosion sensor was loaded into the primer, sprayed out onto AA2024 test panels, and subjected to accelerated corrosion testing. Panels with alodine pretreatment and 150 ppm of the sensor were placed into a salt fog, the Raman ratio was recorded after the coating was applied, and then the panels were placed in the salt fog chamber. Panels were removed from the chamber at approximately weekly intervals and measured with the Raman spectrometer. As the salt fog exposure time increases, the PRR decreases; first sharply, then it plateaus at around 500 h. A second drop and plateau occurred after 1000 h. Visual photographs of the corroding panel show salt deposits developing first, then pitting in the scribe and finally, coating removal towards the end of nearly 1000 h in the ASTM B117 (Figure 5). Measurements of the corrosion sensor were made away from the center scribe on the panel but reflect the corrosion occurring there, as a drop in the PRR as a function of time indicates an increasingly acidic environment between the coating and the metal surface.

**Figure 6:**
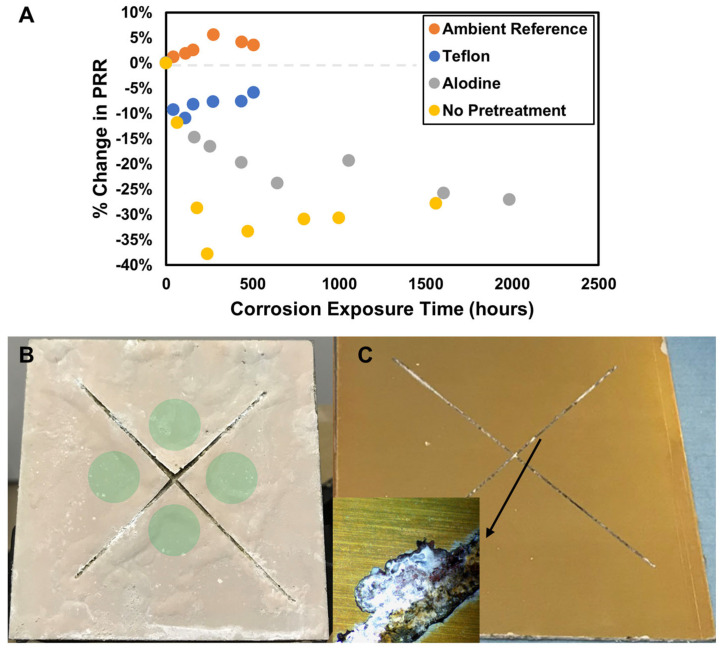
MIL-DTL-53030 primer loaded with corrosion sensor was applied to panels with no pretreatment, or those that had been treated with an alodine conversion coating. (**A**) Raman signal as a function of time in the salt fog. (**B**) A representative “bare” (no pretreatment) panel after 1500 h of exposure to ASTM B117. (**C**) Photographs of the alodine panel, stripped after 2000 h in ASTM B117. (Inset) Deep corrosion damage is present, which was indicated by the corrosion sensor prior to stripping.

Multiple substrates and exposure conditions were tested to verify that the sensor was responding to pH changes resulting from corrosion of the AA2024 metal. A primer loaded with 150 ppm of the sensor was applied to four panels: two with alodine pretreatment, one panel without any pretreatment, and one panel made of Teflon plastic instead of AA2024. Of the two alodine-treated panels, one was kept at ambient conditions, while the other was placed into the salt fog with the remaining substrate effect samples. The PRR was measured as a function of time for all panels, as shown in Figure 6.

The ambient environment panel acted as a negative control. This panel, which was not exposed to salt-fog conditions, did not experience a drop in PRR as the panels in the salt fog chest did. The coated Teflon panel was placed into the salt fog chest to measure the effect of humidity alone on the corrosion sensor in the absence of corrosion. The inorganic alumoxane carrier particle which disperses the 4-MP decorated gold nanoparticle into the primer has many -OH groups across its surface with a pKa ~4.5. The presence of water alone in the coating facilitates an acid/base equilibrium that may slightly alter the pH environment the corrosion sensor experiences. In Figure 6, we can see a small (5–10%) decrease in the PRR from the higher humidity in the B1117 salt fog alone. However, this change is much smaller in comparison to the PRR drop observed with an untreated AA2024 panel (~40%) or an alodine-coated panel (~30%). In addition, the slight decrease in PRR due to humidity does not decrease with time, as the real corrosion PRR does.

Of the two AA2024 test panels in the salt fog, the initial drop in PRR occurred much faster for the panel without any alodine pretreatment. Alodine pretreatments are intended to increase the corrosion protection of the metal. This result suggests that the sensor is responding to more severe and faster initial corrosion on an unprotected panel, and both panels even out to a similar approximate PRR drop of ~30% after 1500 h of salt fog exposure. Photos of these two panels are shown in Figure 6B,C. The no-pretreatment panel shows significant corrosion damage across the entire panel face, with blistering, lifting of the coating near the scribe, and salt deposits. The alodine pretreatment panel showed very little damage at first visual inspection, even after stripping. Closer microscopic inspection showed that the source of the corrosion the sensor was picking up was coming from the scribe region. As shown in Figure 6, there were various spots along the scribe where corrosion had destroyed the alodine conversion coating and started creating deep pits at the edge of the scribe. A separate experiment with a coated steel substrate also showed the fast decrease in PRR with the onset of corrosion (Supplemental Figure S3). 

### 3.5. Detection of Corrosion on AA2024 Curved Panel with Corrosion “Smart” Paint

One benefit of the Raman detection approach is that the interrogation spot can be quite small, regardless of the commercial Raman spectrometer selected. For instance, the diameter of the laser beam in the portable Raman spectrometer used in this work (TSI International) at a stand-off of 7 mm is only 100 µm in diameter. Commercial Raman stand-off Raman spectrometers like the Pendar X10 (Cambridge, MA, USA), operated at stand-off distances of 1–3 ft between the operator and the surface under inspection, still only have analysis spot sizes of ~1 cm in diameter. This means the Raman measurement is more amenable to analyzing complex geometries and curved surfaces, which appear frequently in real aircraft. In addition, surfaces encountered in real-life use cases are likely to be dirty with particulate matter or liquids like hydraulic fluid.

**Figure 7:**
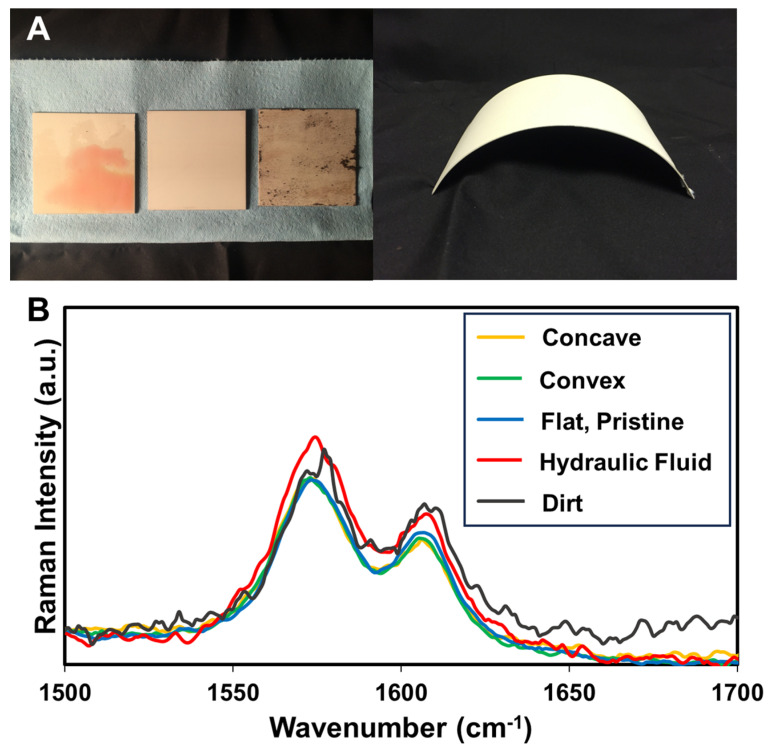
(**A**) Photos of non-ideal test panel surfaces covered in (left to right) hydraulic fluid, pristine, covered in dirt, and curved. (**B**) Raman spectra of the different panel states.

Several test panels were made up with a primer containing the molecular corrosion sensor where the panel shape was varied, or where dirt or hydraulic fluids were applied (Figure 7). Raman spectra were taken for each sample and are shown in Figure 7B. The spectra, with a focus on the regions at 1580 cm^−1^ and 1610 cm^−1^, for the curved panels look practically identical compared to the flat and clean panel, indicating that the curvature makes no difference in the measurement. The Raman spectrum for the dirty panel is a little more noisy, possibly due to dirt particles scattering the incident laser light. Hydraulic fluid is transparent to the Raman wavelength of 785 nm, as are other common aerospace liquids such as aerospace lubricating oil, so other common contaminant fluids are not expected to change the sensor readout. Nonetheless, the PRRs for all samples are within 6% error, indicating a minimum robustness for this corrosion sensing technique.

### 3.6. Comparison of Corrosion Sensor with EIS

Electrochemical impedance spectroscopy (EIS) is a “gold standard” electrochemistry technique for studying the corrosion process. The impedance (real and imaginary components) of the system is mapped from 10 mHz up to 100 kHz. Several features, such as the solution resistance (R_S_) and the double layer capacitance (C_DL_), can be determined directly from the raw data. Other variables such as the polarization resistance (R_P_, also called charge-transfer resistance, R_CT_) can be inferred and then refined by fitting with the appropriate equivalent circuit model. A single 3″× 3″ panel coated with ~1 mill 53030 D was left in an EIS paint cell for 3000+ h. The EIS experiments were recorded at approximately weekly intervals. Each EIS analysis generated one R_CT_ value for that time interval. Plotting all R_CT_ values as a function of time shows a fast decrease and then a plateau as corrosion proceeds. Since the RCT is inversely related to the corrosion current (I_Corr_), the decrease in R_CT_ as a function of time indicates that more current is flowing across the panel face as the corrosion is enhanced from the accelerated test protocol.

**Figure 8:**
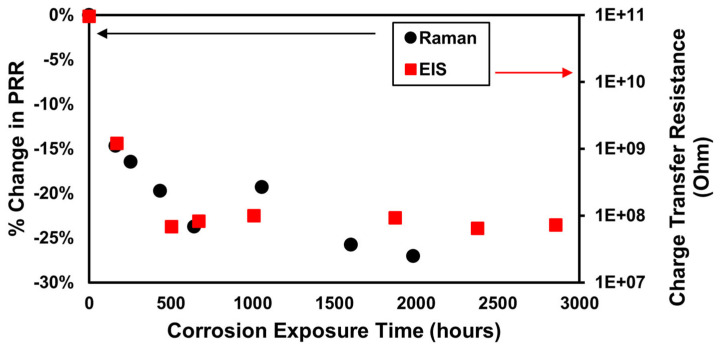
Comparison of accelerated corrosion on a 3″ × 3″ AA-2024 panel coated in MIL-DTL-53030, as assessed by the Raman corrosion sensor or electrical impedance spectroscopy (EIS). The decrease in the charge transfer resistance tracks well with the decrease in the Raman sensor, brought on by a decrease in pH due to active and severe corrosion.

The Raman pH ratio for a sister panel, measured over the same time interval as the EIS data collection, shows the same pattern for aggressive, accelerated corrosion on a 3″ square panel. The ratio drops quickly then reaches a plateau. The origin of the higher [H+] is the dissolution of the Al metal (anodic half-cell). As the I_Corr_ increases, the concentration of [Al^3+^] increases, as does the [H+] (the pH of the solution between the metal and primer decreases). Thus, it makes sense that the change in R_CT_ and the undercoat pH reflected by the Raman sensor are tracked together (Figure 8). The B117 salt fog keeps the panels wet constantly, as does an immersion cell, so the corrosion methods were similar despite the slight temperature difference between the two (35 vs. 25 °C).

### 3.7. Defining Spatial Reporting Region of Corrosion Sensor

The interrogation spot size of the laser from the Raman spectrometer is very small, and the corrosion sensor is well dispersed in the coating. The protons generated from acidic corrosion conditions are incredibly small (ionic radius ~0.88 × 10^−15^ m), so the pH changes from a pit field can move quickly through the coating or between the coating–metal interface. This means that when inspecting an aircraft for corrosion, the operator does not need to measure directly on top of where the corrosion is happening. Rather, active and potentially dangerous corrosion will have an acidic pH zone emanating from it, so this molecular sensor may allow a user to find corrosion by following the trail of decreasing pH signals.

To investigate the size of the “corrosion zone” the sensor could pick up on, we moved from 3 × 3″ test panels to larger 12″ × 12″ coated panels, with the corrosion sensor in the coating. A scribe was placed in the lower right-hand corner of the panel and the Raman signal was measured at specified distances from the center of the scribe, ranging from 2.5″ to 14″ as a function of time in the ASTM B117 accelerated test. A total of 23 analysis areas were drawn on the surface of the panel with a fine-tipped black sharpie to provide a point of reference to make the Raman measurements as close to the same spot as possible at each time interval. Measurements were made approximately weekly (~166 h), resulting in ~30 measurements per measurement area. To simplify the figure and allow easier comparison between the spatial locations on the panel, a line drawn through the data points is used for comparison at each location.

**Figure 9:**
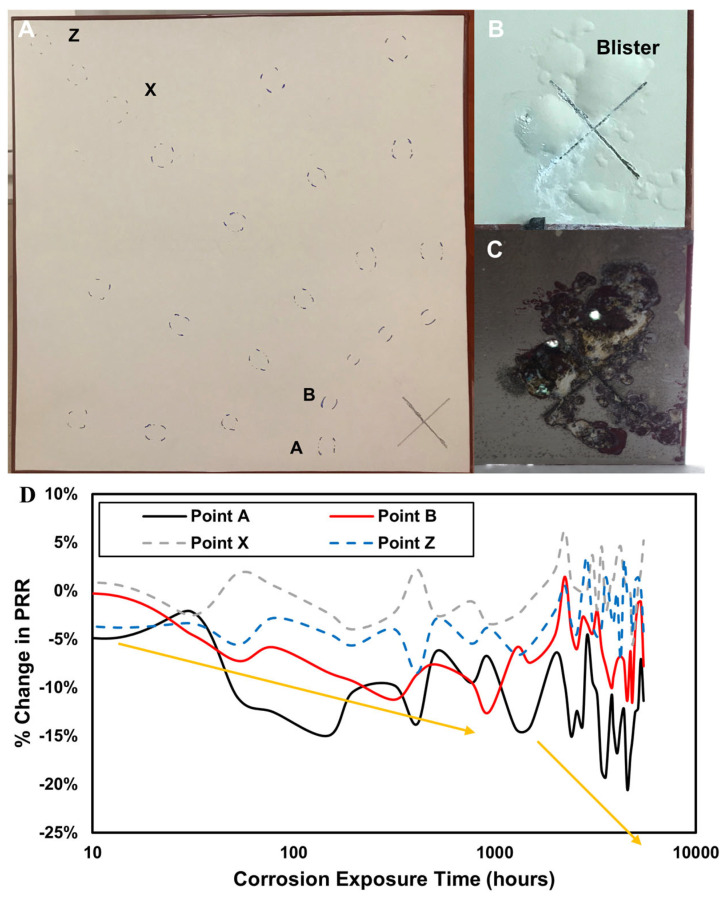
(**A**) 12″ × 12″ panel used for spatial resolution testing. The scribe is in the bottom right and circles are marked with pen on the surface of the panel to measure the same locations at each time point in ASTM B117. (**B**) Zoomed-in scribe after 2500 h in ASTM-B117. (**C**) Zoomed-in scribe after coating was stripped at 6000 h in ASTM B117. Note the holes where it has corroded clean through the panel. (**D**) PRR values at the spots labeled A in the salt fog.

The results for the selected maximum and minimum distance points are shown in Figure 9. Our hypothesis was that a proton “front” would emanate radially from the high-damage location at the scribe. The points at a far distance (points X and Z in the upper left-hand corner) acted as reference points, oscillating between a 0 and 5% PRR change. Once stripped, the far corner showed no damage.

Overall, the panel tested here had exceptionally high primer adhesion. As such, it took a long time to show any visible evidence of corrosion-related damage. Points A and B were closest to the scribe and show another sharp negative slope change starting around 2000 h in the B117 salt fog (Figure 9). Position A of the radius closest to the scribe saw an especially sharp drop in the Raman ratio, indicating a drop in pH brought on by corrosion. Position B also dropped, but not as quickly, and the change was not as large as for position A. Over the course of the salt fog exposure, oscillations were stronger at positions A and B in the first two radii and were weaker or non-existent at the positions in the corner furthest from the scribe (X and Z). Eventually, after ~4500 h in the salt fog, positions X and Z in the opposite corner began to show stronger oscillations (Figure 9), which we interpreted as the condition’s undercoat becoming bad enough that corrosion had reached all the way across the panel.

Measurements were also taken on the blister sections right near the scribe (Figure 9). Though large blisters had grown in around the scribe, no delamination of the primer had been observed. The very large blisters near the scribe were raised and hardened as if they were filled between the metal and the primer. We surmised this to be a buildup of salt and aluminum hydroxide crystals a result of the decreased water access to the scribe.

At 2000 h in the salt fog, we began to track the Raman ratio of the two largest blisters at the top and to the left of the scribe. Measurements of the Raman ratio showed much lower values (50% drop in PRR) at the blister sites compared to the rest of the panel, indicating that corrosion was especially active underneath these blister patches, even though the blisters were not changing visually. Upon stripping the panel (Figure 9), two ~5mm holes located directly under the two large blister patches at the scribe were observed. The sensor had correctly indicated that corrosion was especially bad at these spots. Indeed, it was so corrosive that it led to the complete dissolution of the metal panel at that location.

## 4. Discussion

Particle size and shape can change the SERS enhancement factor and many studies have found that the Raman signal increases with particle size, up to approximately 100 nm [41,42,43]. As such, we targeted 100 nm in our synthesis. Any coinage material will work as the basis of the nanoparticle, however silver and gold provide the best signal enhancement and gold is the best for chemical stability [44]. Many commercial aircraft primers and pretreatments use species with high oxidation-reduction potential, which can spontaneously react with silver given its low redox potential. For example, the reaction between CrVI → CrIII and Ag → Ag+ is spontaneous and precludes the use of silver nanoparticles in legacy chrome-containing coatings.

The use of 4-MP as a pH sensing molecule utilizing SERS has been widely reported in the literature. The peaks that are monitored can vary throughout the literature, but the molecule is consistently found to have a somewhat sinusoidal pH response, becoming less responsive at extreme pH values, with the most linear region being between pH 2 and 6 in the aqueous solution [23,45,46]. In our experiments, it was mostly linear throughout the entire pH range tested in the solution. Once placed into the primer, the sensor is relatively unresponsive between pH 3 and 11, with the best sensitivity between pH 1 and 3. This makes the corrosion sensor very good at detecting sharp pH drops, which are usually associated with severe corrosion. Differences between the pH response in the solution and in the primer are also expected, since the local environment within a primer is complex, and this will be a point of future study.

Extreme pH values are almost always correlated with high corrosion rates [6,7,47]. As such, an indicator that alerts to very high or low pH values is helpful to identify the occurrence of severe corrosion. Thus, we do not expect false positives to be an issue with this sensor. Within the larger area of an aircraft, any spot showing a sharp drop in PRR indicates corrosion occurring within a ~5″ zone of where the measurement is being taken.

A change in the PRR is correlated to a change in pH, but it is necessary to know the starting PRR of the coating when it is first applied. The pH calibration tests were primarily designed to confirm the responsiveness of the sensor to pH changes. The corrosion experiments on multiple substrate types confirmed that the sensor responds to corrosion; therefore, the sensor responds to the pH changes generated during corrosion. Overall, the most severe corrosion was found to be concurrent with PRR values of 1, representing highly acidic values with high concentrations of protons released during pitting corrosion.

The homogenous dispersion of our sensor in the coating, coupled with the ability of very small protons to move through a wetted coated in the ASTM B117, allowed us to attempt a new experiment with a large panel in an attempt to capture the evolution of a corrosion proton front. This initial experiment was the first step in connecting lab-scale accelerated corrosion tests on small panels with larger panels, with the long-term goal of better connecting lab-scale corrosion studies with in-field corrosion environments.

The PRR in the large panel oscillates for all areas. The oscillation occurs along a straight line for the point distal to the corrosion, along a downward trend for the areas nearest to the most active corrosion (i.e., scribe). One contributing factor in this could be the stochastic nature of corrosion. Corrosion is not a linear and consistent process, it is instead highly randomized, with electrochemical corrosion reactions starting and stopping at various locations all over the panel face. Pitting reactions are especially prone to this, and this is the corrosion reaction that generates the most protons. The stochastic nature of corrosion, especially pitting corrosion, is a complicating factor in the modeling of corrosion, as described twenty years ago by Valor [48] and more recently by Wang [49]. Therefore, as pitting is a stochastic process, the measurement of protons/pH could have substantial variability, leading to this oscillation. As discussed earlier, the alumoxane carrier surface around the 4-MP functionalized gold nanoparticles has many -OH groups and a pKa ~4.5. This sets up a second pH equilibrium, which could affect the sensor readout on top of the pH environment being generated by the pitting corrosion. Though we note that pitting corrosion is associated with very acidic pH outside the pKa buffer range, and very low PRRs are always associated with extreme corrosion, improving the carrier particle selection in the future could help clarify data collected in additional experiments probing the localization of the proton front from an active corrosion site on a larger surface. To our knowledge, the experiments here are the first to attempt to measure a “proton front” for corrosion in a coating on a metal surface. Use of the molecular corrosion sensor to establish spatial information on corrosion progress by reading the pH at different locations will be an active avenue in future work.

## 5. Conclusions

Overall, the corrosion sensor developed here works well to identify corrosion occurrence. Consistently, the sensor responds by dropping the PRR, and values near 1 indicate severe levels of corrosion. Batch-to-batch consistency and tuning the signal via the chemistry of 4-MP adsorption and gold nanoparticle morphology, along with exploring new standoff technologies and different primers, are all promising areas for future research to enable the commercial viability of this technology.

From the described experiments and calibrations, we infer a corrosion-related proton front radius of 3″–4″ from the site with the most severe corrosion (the blisters near the scribe). During actual use on an aerospace asset, we envision probing of the surface at regular intervals with a Raman spectrometer. We expect a sensor change anywhere in a diameter of 8″ around active high-rate pitting corrosion, which can then be followed back to the source to determine where the worst corrosion is occurring. Subsequent measurements would follow further decreases in PRR until the area with the lowest PRR is found. This area corresponds to the worst corrosion occurring under the coating, then requires mediation.

## 6. Patents

V. Nguyen, Cook, R.L., Elliott, J.E., Biller, J.R., “Undercoat Corrosion Monitoring Raman Sensor”, 2022. US Patent Application #17/688628.

## Figures and Tables

**Table 1:** ASTM methods for physical property testing.

Physical Property Test	ASTM Reference
Tape adhesion	D3359
Color	D2244
Solvent resistance	D5402
Flexibility	D6905
Hardness	D3363

## Data Availability

Data are available upon request to the corresponding author.

## Supplementary Materials

The following supporting information can be downloaded at: https://www.mdpi.com/article/10.3390/s25010179/s1, Figure S1. Average PSD of the gold nanoparticle suspension. Figure S2. SEM Image of the region interrogated by EDX. Figure S3. Raman ratio as a function of corrosion exposure time for primer coated samples. Table S1. Effects of sensor loading on coating physical properties
