# Supplementary material for: In Situ Raman Spectroscopy for Early Corrosion Detection in Coated AA2024-T3
Source: Sensors (Basel). 2024 Dec 31;25(1):179. doi: 10.3390/s25010179 (PMC11723063; doi:10.3390/s25010179)
Supplement: Supplementary file 1 [file sensors-25-00179-s001.zip › sensors-3346078-supplementary.pdf]

Supplementary Information

"In-Situ Raman Spectroscopy for Early Corrosion Detection in Coated AA2024-T3"

A.K. Delluva et. al.

A. Particle Size Distribution

The data shown in SI1 is the average of 3 runs.

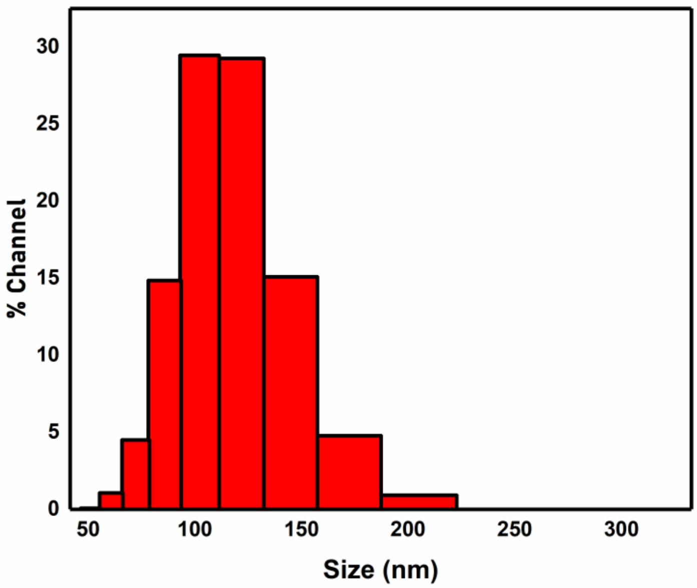

Figure S1. Average PSD of the gold nanoparticle suspension prior to being incorporated into the sensor powder.

B. Presence of gold nanoparticles on alumoxane carrier particles

While obtaining SEM images of the sensor powder, EDX analysis was run on the bright white spots to confirm that they are composed of gold. Samples were coated with carbon to not interfere with the elemental analysis. The results of a selected spot run of the analysis is shown in SI2.The point was set on a bright white, gold agglomeration spot. The analysis does show a prominent presence of gold. The results are not purely gold, given the low resolution/large analysis spot size. Aluminum and oxygen are also present, representative of the carrier particle, as well as carbon, representative of the coating. We believe the Mo presence to be an artifact, as it is present on the shoulder of the gold peak.

A

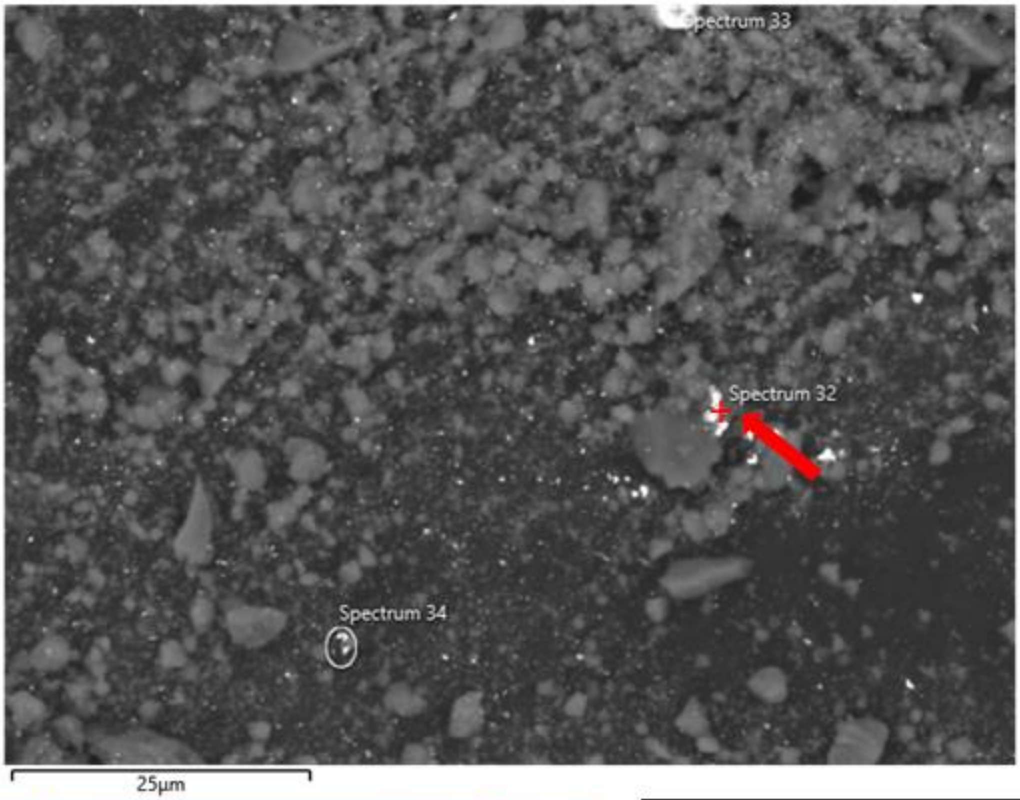

B

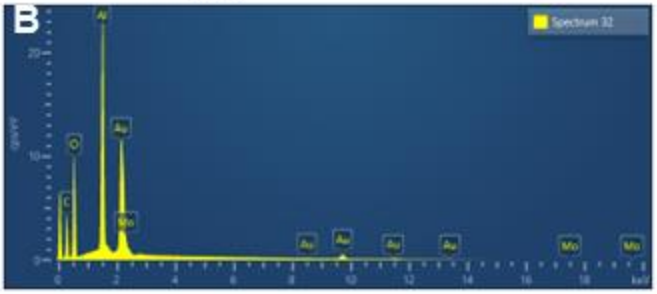

C

Spectrum 32		
**Element**	Line Type	Weight %
**C**	K series	28.99
**O**	K series	26.24
**Au**	M series	27.28
**Al**	K series	14.70
**Mo**	L series	2.80
**Total**		100.00

Figure S2. (A) SEM image of the region interrogated by EDX. The data in (B) and (C) was taken from the spectrum highlighted. (B) Collected energy bands, identifying present elements. (C) Calculated weight percent of identified elements.

C. Substrate Effects

Applicability to a wide range of systems is beneficial, so a different substrate (1018 Steel) was tested, as well as a sample with topcoat. The steel substrate showed similar corrosion behavior as the aluminum panel (S3). This is in alignment with corrosion on metallic substrates releasing protons and becoming acidic. The primary reaction for steel is Fe → Fe.

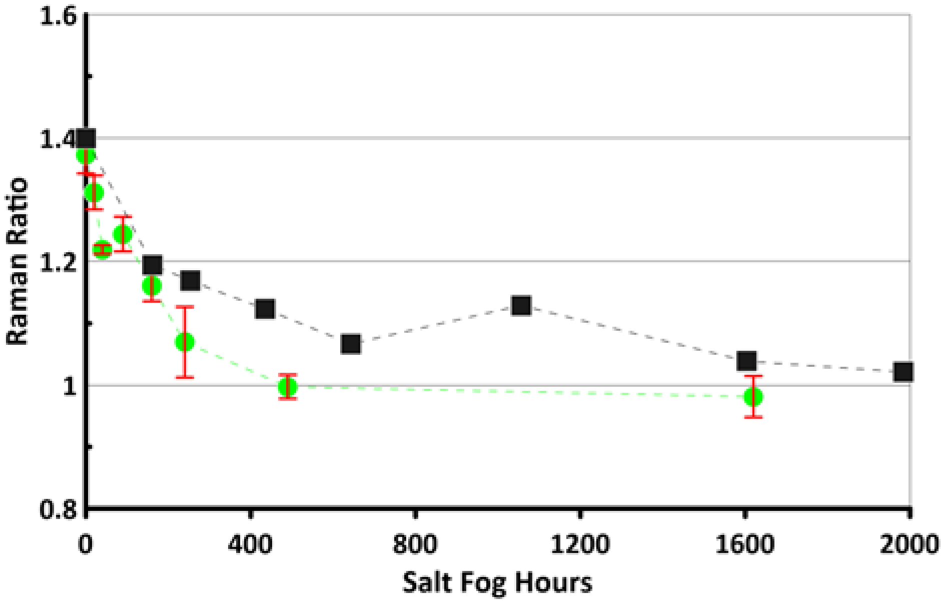

Figure S3. Raman ratio as a function of corrosion exposure time for primer-coated samples. Black squares are the aluminum substrate, and green dots are the steel substrate sample.

Table S1. Effect of sensor loading on coating physical properties

	Gloss			Color			Tape Adhesion	Pencil Hardness	Chemical Resistance
**Coating**	20°	60°	85°	L	a	b	ASTM 3359B	ASTM 3363	MEK Double Rub
**MIL-DTL-53030D (Unmodified)**	0.9	2.2	2.4	81.06	-0.8	9.55	5A	7H	
**MIL-DTL-53030D (150 ppm Sensor)**	0.9	1.9	2.2	80.97	-0.31	9.75	5A	7H	>200
